# Effects of Structured Lifestyle Education Program for Individuals With Increased Cardiovascular Risk Associated With Educational Level and Socioeconomic Area

**DOI:** 10.1177/1559827620951143

**Published:** 2020-08-29

**Authors:** Matthias Lidin, Mai-Lis Hellenius, Monica Rydell Karlsson, Elin Ekblom-Bak

**Affiliations:** Department of Medicine, Karolinska Institutet, Karolinska University Hospital, Solna, Stockholm, Sweden; Department of Medicine, Karolinska Institutet, Karolinska University Hospital, Solna, Stockholm, Sweden; Department of Clinical Sciences, Karolinska Institutet, Danderyd Hospital, Stockholm, Sweden; Ersta Sköndal Bräcke University College, Stockholm, Sweden; The Swedish School of Sport and Health Sciences, Stockholm, Sweden

**Keywords:** lifestyle habits, cardiovascular risk, education level, socialeconomic areas, quality of life

## Abstract

*Background.* Differences in socioeconomic status contribute to inequalities in lifestyle habits and burden of noncommunicable diseases. We aimed to examine how the effects of a 1-year structured lifestyle education program associate with the participant’s educational level and socioeconomic area (SEA) of residence. *Methods.* One hundred individuals (64% women) with high cardiovascular risk were included. Education level (nonuniversity vs university degree) was self-reported and SEA (low vs high) defined by living in different SEAs. Lifestyle habits and quality of life were self-reported, cardiovascular risk factors and Framingham 10-year cardiovascular disease risk were measured at baseline and after 1 year. *Results.* Sedentary behavior decreased in both nonuniversity degree and low SEA group over 1 year, with a significantly greater improvement in daily activity behavior in low- compared with high-SEA group. Abdominal obesity decreased significantly more in the nonuniversity compared with the university degree group. Cardiovascular risk and quality of life improved in all groups, however, with greater discrimination when using educational level as the dichotomization variable. *Conclusion.* The results are clinically and significantly relevant, suggesting that low socioeconomic status measured both as educational level and SEA are no barriers for changing unhealthy lifestyle habits and decreasing cardiovascular risk after participation in a lifestyle program.

‘Inequalities in healthy lifestyle behaviors between different socioeconomic subgroups may be reduced after participation in structured interventions . . .’

Life expectancy is increasing generally, however, with large variations between different socioeconomic groups.^1,2^ Two commonly used measures for definition of socioeconomic groups are educational level and area of residence, and both low educational level and low socioeconomic area (SEA) have been associated with shorter life expectancy in the Swedish population.^3,4^ These inequalities in life expectancy are driven by, for example, a higher prevalence of obesity, hypertension, diabetes type 2, and increased cardiovascular risk,^5-10^ which are largely attributed to an unhealthy lifestyle and adverse living conditions in these subgroups.^11^ In a nationwide survey in Swedish adults from 2016, participants with lower educational level were more often daily smokers, exercised less, and had lower daily intake of fruit and vegetables.^3^ Moreover, low educational level and low SEA have also been associated with a lower health-related quality of life.^12-14^

Inequalities in healthy lifestyle behaviors between different socioeconomic subgroups may be reduced after participation in structured interventions, as more beneficial effects have been reported in low socioeconomic groups.^15,16^ In a previous study investigating the influence of demographic factors and lifestyle advice given in a primary care setting, 39% of the participants reported changes in lifestyle habits due to the lifestyle advice given, interestingly more often in individuals with low education, as well as among old adults and among men.^15^ Although previous studies have demonstrated important differences in lifestyle habits and disease risk between educational levels and SEA of residence, together with some promising results from intervention studies to offset these inequalities, there is still a need for a greater understanding on how inequalities in health can be counteracted. Also, comparisons between different proxy measures used to define socioeconomic grouping is limited. We have previously reported significant positive changes in multiple unhealthy lifestyle habits, including increased physical activity (PA), decreased time spent sedentary, a more healthy food pattern and decreased alcohol intake, as well as reduced cardiovascular risk in individuals with high cardiovascular risk after participation in a 1-year structured lifestyle education program.^17,18^ However, the influence of the participant’s educational level and SEA of residence was not illuminated.

Therefore, the aim of the present study was to examine how the effects of a 1-year structured education lifestyle program on change in unhealthy lifestyle habits, cardiovascular risk, and quality of life associated with the participant’s educational level and SEA of residence. We hypothesized that participants with low educational level (no university degree) and living in low SEA would have more adverse lifestyle habits, higher cardiovascular risk, and lower quality of life, but experience greater beneficial changes over 1 year, compared with their high educational (university degree) and SEA counterparts.

## Methods

In 2008, a 1-year structured lifestyle program with a multidisciplinary approach for individuals with high cardiovascular risk was initiated at the cardiology unit of Karolinska University Hospital in Stockholm, Sweden. The focus of the program was to target individuals with unhealthy lifestyle habits and a subsequent increased cardiovascular disease (CVD) risk, referred to the program by their physician from either primary or hospital care. The primary goal of the program was to guide the individuals to improve lifestyle habits. Inclusion criteria were men and women ≥18 years presenting at least 3 of the following risk factors; physical inactivity, unhealthy food habits, present smoking, risky consumption of alcohol, high perceived stress, (general overweight) elevated body mass index, abdominal obesity, dyslipidemia, high blood pressure, insulin resistance, type 2 diabetes, or previous CVD. Exclusion criteria were an inability to understand the Swedish language or to attend the entire program, alcohol addiction, and psychiatric diagnoses. Between 2008 and 2014, 140 men and women were enrolled in the program. Twenty-four were excluded according to exclusion criteria, and 16 declined participation, leaving 100 individuals for the present study. This study was conducted as a pilot study due to the small sample size which limits the power for subgroups analyses.

### The Intervention Program

**Figure 1.**
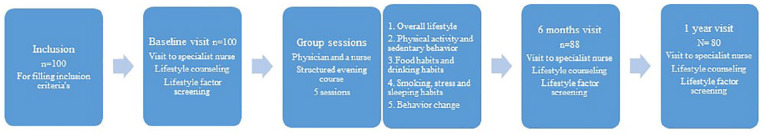
Flowchart of the structured lifestyle education program over 1 year.

The patient education program has previously been described.^18^ In short, the program comprised both individual and group visits. At baseline, after 6 months, and after 1 year, the participants met with a nurse for a health check-up and an additional dialogue using a person-centered approach with motivational technique to support behavioral change. At baseline, the participant also received a prescription of physical activity and a pedometer, and was offered participation in five educational group sessions led by the nurse and a physician with a focus on (*a*) overall lifestyle and health, (*b*) physical activity and sedentary behavior, (*c*) dietary habits and use of alcohol and tobacco, (*d*) stress and sleeping habits, and (*e*) behavioral change (Figure 1).

### Lifestyle Habits and Quality of Life

Lifestyle habits, living conditions and quality of life were obtained by questionnaires at all 3 visits, which has been described in detail previously.^17^ Time spent sedentary was reported in hours and minutes using the International Physical Activity Questionnaire (IPAQ) Short Form.^19^ Daily activity was dichotomized into ≥30 minutes per day or less, and exercise habits into ≥1 hour per week or less. Smoking was dichotomized into daily smoking or not, and risk consumption of alcohol was based on sex-specific evaluations of frequency and quantity of alcohol intake.^20^ Dietary habits were assessed by 14 validated questions, covering, for example, daily intake of vegetables, quality of fat and extra calories from snacks. Quality of life was based on the Goteborg Quality of life instrument (GQL), validated for patients with high cardiovascular risk,^21^ which consists of 16 questions divided in to 3 subscores of well-being; social well-being (including questions regarding home-situation, housing, work, economy, leisure time), mental well-being (including questions regarding mood, energy, patience, self-esteem, sleep), and physical well-being (including questions regarding health, fitness, memory, appetite, vision, and hearing).

### Cardiovascular Risk

Waist circumference was measured to the nearest 0.5 cm in a standing position, midway between the lower rib margin and the iliac crest. Systolic and diastolic blood pressure (BP, in mm Hg) was measured in seated position after a 10-minute rest using standard auscultatory method with a cuff. A blood sample was drawn from an antecubital vein after overnight fasting, and total s-cholesterol (mmol/L) and s-high-density lipoprotein (HDL, mmol/L) were assessed by standard methods according to local laboratory routines at Karolinska University Hospital. Framingham 10-year risk prediction model was used to assess cardiovascular risk based on age, smoking, systolic BP, total cholesterol, HDL, and prevalence of type 2 diabetes.^22^ The prediction model estimates a 10-year probability of developing a CVD (in %). Probabilities were obtained in the total study population, as well as with regard to previous diagnosed CVD or not. Previous CVD and present type 2 diabetes diagnoses were derived from the participants’ medical journal.

### Educational Level and Socioeconomic Area of Residence

Educational level was self-reported and categorized into university degree or nonuniversity degree. The participants’ demographic data including addresses were obtained from medical journals. Classification into low or high SEA was based on calculation of median income in Sweden by official statistics from Statistics Sweden (SCB).^23^ Low SEA was defined as areas with median income ≤29 300 Swedish crowns, and high SEA as areas with a median income of more than 29 300 Swedish crowns. Each participant was then identified as resident of either a low or high SEA according to the mean income in the postcode area of residence.^24^

### Ethical Considerations

To change unhealthy lifestyle habits in a health care setting could be a very vulnerable situation and it is important that the participant is met respectfully, having the participant’s autonomy in mind when involving them in their own treatment. In this study, we strived to achieve this through the person-centered approach, and working in line with Good Clinical Practice guidelines. All participants provided a written consent. The study was approved by the local ethics committee in Stockholm DRN 2015/494-31/2. The study was registered at www.clinical-trials.gov (ClinicalTrial.gov ID: NCT02744157).

### Statistical Analysis

Data were checked for normality using Shapiro-Wilk test. The majority of variables were skewed, thereby data are presented as median (quartiles 1 and 3). Intention to treat approach was used, and hence last observation carried forward or backward was used for missing data. Differences in proportion of unhealthy lifestyle habits (*a*) at baseline between university and nonuniversity degree participants, and participants living in low and high SEA of residence, respectively; (*b*) delta change of proportions within each group; and (*c*) comparisons of delta change between groups, were tested by calculating the raw difference and a 95% confidence interval for the difference. For the skewed continuous data (Framingham risk score and quality of life), differences at baseline between groups as well as comparisons of delta change over 1 year between groups were calculated using Mann-Whitney *U* test, with median difference and 95% confidence interval presented. To test for significant delta change within each group over 1 year, Wilcoxon matched test was used. Significance level was set to *P* < .05. Statistical analyses were performed using SPSS (version 24) and Confidence Interval Analysis (version 2.0.0).

## Results

One hundred individuals were included in the study (n = 64 women), with a mean age of 58 ± 11 years. Fifty-three participants (53%) reported nonuniversity degree and 59 (59%) were defined as living in low SEA. While there were no sex differences between the nonuniversity and university group, or between the low and high SEA group, higher prevalence’s of type 2 diabetes (30% vs 11%) and CVD (43% vs 28%) were observed in the nonuniversity group compared with the university group. No differences were found between the SEA groups.

### Unhealthy Lifestyle Habits in Relation to Educational Level

Except for exercise habits, the baseline differences in prevalence of unhealthy lifestyle factors varied marginally between the nonuniversity and university degree participants. While sedentary risk behavior decreased significantly in the nonuniversity degree participants, the decreases for the other lifestyle variables seemed to be more pronounced in university degree participants (nonsignificant). There were no significant difference in changes over 1 year between the 2 groups.

### Cardiovascular Risk and Quality of Life in Relation to Educational Level

At baseline, the prevalence of abdominal obesity was significantly higher in nonuniversity degree participants compared with university degree participants (89% vs 60%) (Table 1). A significantly greater decrease (−17%) was noted in the nonuniversity degree participants over 1 year, compared with university degree participants (−4%). Nonuniversity degree participants had a significantly higher 10-year cardiovascular risk at baseline (Table 2). The 10-year cardiovascular risk decreased significantly in both nonuniversity and university degree groups over 1 year. When divided into previous or nonprevious CVD, decreases were seen in both previous and nonprevious CVD participants with nonuniversity degree, but only in nonprevious CVD participants with university degree. All quality of life subscores of well-being were similar in both groups at baseline. Physical well-being increased significantly in the university degree group over 1 year, with no such change in the nonuniversity degree group. Mental well-being increased in both groups, with no significant change of social well-being.

**Table 1.** Proportions of Participants With Unhealthy Lifestyle Habits and Abdominal Obesity at Baseline and 1 Year, and Changes Over 1 Year, in Nonuniversity (n = 53) and University (n = 47) Degree Participants.^a^

		Non-university degree				University degree			Difference in change over 1 year between groups
			Baseline	One year	Change over 1 year	Baseline	One year	Change over 1 year	
					Proportion difference (95% CI)			Proportion difference (95% CI)	Proportion difference (95% CI)
*Unhealthy lifestyle habits*									
High sedentary time	n = 45	47% (21)	31 (14)	−16% (−28 to −3)	n = 45	44% (20)	33% (15)	−11% (−26 to 5)	−4% (−19 to 10)
Low daily activity	n = 52	46% (24)	44% (23)	−2% (−15 to 11)	n = 47	45% (21)	36% (17)	−9% (−23 to 6)	7% (−3 to 18)
Low exercise	n = 43	86% (37)	77% (33)	−9% (−24 to 6)	n = 38	71% (27)	58% (22)	−13% (−29 to 4)	4% (−11 to 19)
Smoking	n = 51	12% (6)	12% (6)	0% (−8 to 8)	n = 47	9% (4)	6% (3)	−2% (−11 to 6)	2% (−5 to 11)
Alcohol risk consumption	n = 50	22% (11)	22% (11)	0% (−11 to 11)	n = 45	22% (10)	18% (8)	−4% (−17 to 7)	4% (−3 to 15)
Low intake of vegetables	n = 52	87% (45)	81% (42)	−6% (−16 to 4)	n = 47	81% (38)	70% (33)	−11% (−24 to 2)	5% (−7 to 17)
High intake of extra calories	n = 49	37% (18)	33% (16)	−4% (−19 to 11)	n = 47	38% (15)	28% (11)	−10% (−22 to 3)	6% (−6 to 19)
*Abdominal obesity*									
High waist circumference (>100 cm)	n = 53	89% (47)^b^	72% (38)	−17% (−29 to −6)	n = 47	60% (28)	55% (26)	−4% (−16 to 8)	−13% (−25 to −0.1)

a Data presented as % (n) or median (Q1 to Q3).

b Significant group difference at baseline, *P* < .05.

**Table 2.** Proportions of Participants’ Cardiovascular Risk and Quality of Life at Baseline and 1 Year, and Changes Over 1 Year, in Nonuniversity (n = 53) and University (n = 47) Degree Participants.^a^

	Nonuniversity degree					University degree			
			Baseline	One year	Change over 1 year	Baseline	One year	Change over 1 year	Difference in change over 1 year between groups
					Median difference			Median difference	*P* value for median difference between groups
*Cardiovascular risk*									
Framingham 10-year risk, all (%)	n = 53	15.9 (10.9-26.4)^b^	15.6 (8.0-20.0)	−2.0^c^	n = 47	11.7 (4.5-21.6)	10.0 (4.5-21.5)	−0.6^c^	.13
Framingham 10-year risk, previous CVD (%)	n = 23	25.3 (15.9-30.0)	18.5 (13.7-29.4)	−1.7^c^	n = 13	15.6 (9.9-29.7)	18.4 (7.0-28.5)	0.0	.30
Framingham 10-year risk, non-previous CVD (%)	n = 30	15.6 (9.2-19.3)	10.9 (6.3-15.9)	−2.2^c^	n = 34	8.6 (3.9-19.3)	7.7 (3.8-15.7)	−0.9^c^	.26
*Quality of life*									
Physical well-being	n = 50	27.0 (21.0-30.5)	26.5 (23.0-33.0)	1.0	n = 46	26.5 (21.8-31.3)	28.0 (24.0-33.3)	1.0^c^	.24
Mental well-being	n = 49	21.0 (16.0-27.0)	25.0 (19.0-28.0)	1.0^c^	n = 46	22.5 (17.0-27.0)	24.0 (20.0-29.0)	1.0^c^	.97
Social well-being	n = 30	25.0 (20.8-29.3)	27.0 (23.0-31.3)	1.5	n = 40	27.5 (23.0-29.0)	26.5 (23.0-30.0)	0.5	.33

Abbreviation: CVD, cardiovascular disease.

a Data presented as % (n) or median (Q1 to Q3).

b Significant group difference at baseline, *P* < .05.

c Significant delta change over 1 year within group, *P* < .05.

### Unhealthy Lifestyle Habits in Relation to Socioeconomic Area

Significantly fewer individuals from the low-SEA group exercised regularly at baseline, with lower daily activity but also lower intake of extra calories compared with the high-SEA individuals (Table 3). Although sedentary risk behavior was prevalent to a similar extent at baseline, the proportion decreased significantly only in the low-SEA group. Similar trends were seen for risk behavior of low levels of regular exercise. Comparing change over 1 year, participants in the low-SEA group improved daily activity habits significantly more compared with high-SEA group, and a trend toward positive change of exercise habits was noted.

**Table 3.** Proportions of Participants With Unhealthy Lifestyle Habits and Abdominal Obesity at Baseline and 1 Year, and Changes Over 1 Year, in Low (n = 59) Versus High (n = 41) Socioeconomic Area of Residence.^a^

		Low socioeconomic area				High socioeconomic area			Difference in change over 1 year between groups
			Baseline	One year	Change over 1 year	Baseline	One year	Change over 1 year	
					Proportion difference (95% CI)			Proportion difference (95% CI)	Proportion difference (95% CI)
*Unhealthy lifestyle habits*									
High sedentary time	n = 52	47% (24)	29% (15)	−17% (−29 to −5)	n = 38	45% (17)	37% (14)	−8% (−25 to 9)	−9% (−23 to 6)
Low daily activity	n = 58	50% (29)	40% (23)	−10% (−23 to 3)	n = 41	39% (16)	42% (17)	2% (−11 to 15)	−12% (−23 to −2)
Low exercise	n = 49	88% (43)^b^	71% (35)	−16% (−30 to −2)	n = 32	66% (21)	63% (20)	−3% (−22 to 16)	−13% (−26 to 2)
Smoking	n = 57	12% (7)	12% (7)	0% (−8 to 8)	n = 41	7% (3)	5% (2)	−2% (−13 to 7)	2% (−4 to 13)
Alcohol risk consumption	n = 56	23% (13)	23% (13)	0% (−11 to 11)	n = 39	20% (8)	15% (6)	−5% (−17 to 7)	5% (−2 to 17)
Low intake of vegetables	n = 59	83% (49)	76% (45)	−7% (−17 to 3)	n = 40	85% (34)	73% (30)	−10% (−23 to 3)	3% (−8 to 17)
High intake of extra calories	n = 56	34% (19)	30% (17)	−4% (−17 to 10)	n = 33	42% (14)	30% (10)	−12% (−26 to 3)	9% (−3 to 24)
*Abdominal obesity*									
High waist circumference (>100 cm)	n = 59	83% (50)^b^	69% (41)	−15% (−26 to −4)	n = 41	61% (25)	56% (23)	−5% (−17 to 7)	−10% (−22 to 3)

a Data presented as % (n) or median (Q1 to Q3).

b Significant group difference at baseline, *P* < .05.

### Cardiovascular Risk and Quality of Life in Relation to Socioeconomic Area

The low-SEA group had a significantly higher proportion of participants with abdominal obesity at baseline compared with the high-SEA group (83% vs 61%) (Table 3), with a significant decrease in the number of individuals with high waist circumference over 1 year (−15%) in the low-SEA group. Total Framingham risk were significant reduced in both groups (Table 4). Divided into subgroups of previous CVD or nonprevious CVD, significant improvements were only present in nonprevious CVD participants. Physical and social well-being were lower in low-SEA group at baseline, with significant improvements in physical and mental well-being only in the low-SEA group.

**Table 4.** Proportions of Participants’ Cardiovascular Risk and Quality of Life at Baseline and 1 Year, and Changes Over 1 Year, in Low (n = 59) Versus High (n = 41) Socioeconomic Area of Residence.^a^

			Low socioeconomic area			High socioeconomic area			Difference in change over 1 year between groups
			Baseline	One year	Change over 1 year	Baseline	One year	Change over 1 year	
					Median difference			Median difference	*P* value for median difference between groups
*Cardiovascular risk*									
Framingham 10-year risk, all (%)	n = 59	15.9 (8.6-25.3)	15.6 (6.3-24.8)	0.0^b^	n = 41	13.7 (6.3-13.7)	11.2 (5.5-18.4)	−2.0^b^	.17
Framingham 10-year risk, previous CVD (%)	n = 23	25.3 (15.9-30.0)	21.6 (15.9-30.0)	0.0	n = 13	15.9 (11.5-30.0)	15.9 (8.4-20.0)	−2.9	.62
Framingham 10-year risk, non-previous CVD (%)	n = 36	13.7 (6.6-18.5)	10.0 (5.3-15.9)	−1.1^b^	n = 28	12.5 (4.3-24.4)	9.0 (3.9-15.6)	−1.3^b^	.15
*Quality of life*									
Physical well-being	n = 58	26.0 (21.0-29.0)^c^	26.5 (23.0-32.3)	2.0^b^	n = 38	29.0 (22.8-33.3)	29.0 (25.0-34.3)	1.0	.61
Mental well-being	n = 58	21.0 (17.0-25.0)	24.0 (19.0-28.0)	1.0^b^	n = 37	25.0 (16.0-29.0)	25.0 (20.5-30.0)	0.0	.25
Social well-being	n = 37	25.0 (20.0-28.0)^c^	25.0 (22.5-28.5)	0.0	n = 33	29.0 (23.0-31.0)	29.0 (24.0-32.0)	1.0	.70

Abbreviation: CVD, cardiovascular disease.

a Data presented as % (n) or median (Q1 to Q3).

b Significant delta change over 1 year within group, *P* < .05.

c Significant group difference at baseline, *P* < .05.

## Discussion

The aim of the present study was to examine how the effects of a 1-year education lifestyle program on changes in unhealthy lifestyle habits, cardiovascular risk and quality of life, are associated with the participant’s educational level and SEA of residence. We found different outcomes regarding both baseline levels and changes in lifestyle habits, waist circumference, cardiovascular risk and quality of life, when educational level and socioeconomic area of residence was taken into account.

### Unhealthy Lifestyle Habits

Significant beneficial changes of sedentary risk behavior were seen in nonuniversity degree and low-SEA participants over 1 year, with a significantly greater improvement in daily activity in the low-SEA group compared with the high-SEA group. Previous research reports that individuals with low education and living in low SEA of residence have a more unhealthy lifestyle pattern, with a subsequent increased cardiovascular risk, compared to individuals with high education and living in high SEA.^9,16,25^ It has been suggested that health-related advices are interpreted and admitted differently by different social class groups, and that individuals with higher education are more likely to modify their diets, give up smoking, and take up healthy physical activities.^26^ One explanation could be different abilities to search for evidence-based facts. Individuals with a higher level of education might know how to search and find relevant information and the level of health literacy is important in this regard.^27,28^ The more pronounced changes in the nonuniversity degree and low-SEA participants in the present study might be due to the person-centered approach during the individual visits, giving an opportunity to individualize the support for behavioral change, which has previously been suggested to reduce inequalities regarding information uptake and health outcomes in individuals with high cardiovascular risk.^29^ Adequate prerequisites to be active is also highly relevant for behavioral change, and may differ between low and high SEA of residence. A British observational study from Scotland identified that deprived areas or low SEA of residence had fewer formal resources for healthy physical recreation, for example, fewer safe open green spaces where people could walk, jog, or cycle, and fewer sports centers. Moreover, fewer residents had access to cars, and public transport routes were sparser and less frequent, making it harder for people to travel elsewhere to use such facilities.^26^ Based on the results with improved sedentary and exercise risk behaviors in participants living in low SEA of residence, our program and its structure may be helpful when planning interventions to increase physical activity in these areas.

There were no significant differences between the groups for the other lifestyle risk behaviors, including smoking, risk consumption of alcohol, and unhealthy dietary habits.

### Cardiovascular Risk

Participants with nonuniversity degree had a significantly higher prevalence of diagnosed type 2 diabetes and CVD compared to participants with university degree education which is in line with previous studies.^6^ Abdominal obesity decreased in all groups, with a significant greater decrease in nonuniversity degree participants compared to university degree participants over 1 year. This may partly be explained by significantly higher waist circumference at baseline, with the effect of regression toward the mean. Still, abdominal obesity is a strong risk factor and a mediator for CVD for individuals with low education and living in low-SEA areas.^10,30^ Factors like walkability and the community food environment has been shown to be important for the individual’s ability for self-care, and several studies have shown that barriers to physical activity and healthy food alternatives in the community can lead to increased body mass index and obesity.^31^

Participants with nonuniversity degree had significantly higher Framingham score compared to participants with university education at baseline. However, significant beneficial changes in total Framingham risk score were noted in both nonuniversity and university, as well as low and high SEA of residence participants over 1 year. Our results are in line with other previous studies concluding that education level is no obstacle for achieving changes in cardiovascular risk in lifestyle intervention programs.^15^ For participants with previous CVD, a significant reduction was observed in the nonuniversity degree group. In EUROSPIRE IV, a cross-sectional survey from 24 European countries, significant differences were shown regarding reaching treatment targets depending on the patient’s educational level in cardiovascular secondary prevention. Participants with low education had significantly poorer lifestyle habits pattern after treatment than participants with higher education.^32^

For participants with no previous CVD, significant reductions were seen in all subgroups. Similar results has been shown in previous studies, where the largest changes were seen in individuals with low education and living in low SEA areas, shown by higher absolute numbers of prevented deaths among the groups with low or middle education.^15^ On the contrary, in the North Karelia Project, greater changes were reported in the higher social economical groups.^33^ As our program showed effects on Framingham score in both participants with CVD and without previous CVD, the results from this study with positive changes in lifestyle habits, cardiovascular risk and quality of life may indicate that this type of structured education program focusing on both primary and secondary prevention, on lifestyle rather than the disease, and with individualized risk management, is a feasible and suitable approach for individuals with both low and high education level and regardless of which SEA they live in.

### Quality of Life

Physical well-being score increased in both the university education group and the low SEA group. In mental well-being score a significant increase in both the university education group and no university education group was noted, this was even seen in the low SEA group. An increased level of self-rated health is shown to be strongly correlated to reduced mortality.^34,35^ The significantly increased physical well-being score may, at least in part, be explained by the increase in physical activity. The mental well-being score may be explained not only by increased physical activity but also from being in a lifestyle program getting help and support finding new ways toward a healthier lifestyle.

### Variation in Results Depending on Different Measures for Socioeconomic Status

Comparisons between different measures used to define socioeconomic grouping is limited, and there might be a variation in such results depending on which measure that is used. In the present study, greater discrepancy between the groups were seen for changes in sedentary and low exercise risk behavior after dichotomization according to SEA of residence compared with education level. While patterns and changes were similar when evaluating abdominal obesity and cardiovascular risk regardless measure of socioeconomic status used, stratification according to university degree or not revealed different patterns of change over one year for the quality of life variables, compared with similar analyses after stratification into low and high SEA of residence. Hence, the present results suggest that future analyses should take into account which measure of socioeconomic status that is used, especially when evaluating changes of physical activity and quality of life variables.

### Strengths and Limitations

Few studies have investigated how inequalities in health can be counteracted in clinical practice and the knowledge is scares regarding the influence of different socioeconomic factors in cardiovascular intervention programs combining primary and secondary prevention. Our study might add new knowledge to this aspect. The fact that the study is not a randomized controlled trial might be a limitation. However, studies on implementation of evidence-based lifestyle interventions for cardiovascular prevention in clinical practice is increasingly requested. In this respect, while randomized controlled studies have high internal validity, our study has a high external validity. Individuals who participated in the program might be more motivated, which may limit the generalizability. The small sample sizes in the subgroup analyzes, is another limitation potentially influencing the power of the analyses. A strength of this study is the long-term follow-up of 1 year. Another strength is the use of a person-centered approach as a key-components to adapt the education program to each individual’s prerequisites and level of preknowledge, and to achieve shared decision made goals. We hope that our experiences and results can add to the current knowledge on how education level and living in different SEA influence individuals participating in a lifestyle program in everyday clinical practice.

## Conclusion

Low educational level measured by university degree or not and living in low SEA can imply a higher burden of unhealthy lifestyle habits and higher CVD risk. This study presents significant and clinical relevant results suggesting that these factors are no barriers for changing unhealthy lifestyle habits and decreasing cardiovascular risk after participation in a structured lifestyle program.
